# Optimization of recombinant antibody production based on the vector design and the level of metabolites for generation of Ig- producing stable cell lines

**DOI:** 10.1186/s43141-023-00474-0

**Published:** 2023-02-22

**Authors:** V. A. Toporova, V. V. Argentova, T. K. Aliev, A. A. Panina, D. A. Dolgikh, M. P. Kirpichnikov

**Affiliations:** 1 Shemyakin-Ovchinnikov Institute of Bioorganic Chemistry, Russian Academy of Sciences, Miklukho-Maklaya ul. 16/10, GSP-7, Moscow, 117997 Russia; 2 Department of Bioengineering, Biology Faculty, Lomonosov Moscow State University, Leninskiye gory 1-12, Moscow, 119234 Russia; 3 Department of Chemical Enzymology, School of Chemistry, Lomonosov Moscow State University, Leninskiye gory 1–3, Moscow, 119234 Russia

**Keywords:** Bipromoter/bicistronic plasmid, Expression vector, Level metabolites, mAbs production, Stable cell lines

## Abstract

**Background:**

The biopharmaceutical industry is significantly growing worldwide, and the Chinese hamster ovary (CHO) cells are used as a main expression host for the production of recombinant monoclonal antibodies. Various metabolic engineering approaches have been investigated to generate cell lines with improved metabolic characteristics for increasing longevity and mAb production. A novel cell culture method based on the 2-stage selection makes it possible to develop a stable cell line with high-quality mAb production.

**Results:**

We have constructed several design options of mammalian expression vectors for the high production of recombinant human IgG antibodies. Versions for bipromoter and bicistronic expression plasmids different in promoter orientation and cistron arrangements were generated.

The aim of the work presented here was to assess a high-throughput mAb production system that integrates the advantages of high-efficiency cloning and stable cell clones to stage strategy selection reducing the time and effort required to express therapeutic monoclonal mAbs.

Development of a stable cell line using bicistronic construct with EMCV IRES-long link gave an advantage in high mAb expression and long-term stability. Two-stage selection strategies allowed the elimination of low-producer clones by using metabolic level intensity to estimate the IgG production in the early steps of selection. The practical application of the new method allows to reduce time and costs during stable cell line development.

## Background

The generation of mAb high-producing cell clones is based on a high-expressing vector and optimized selection of stable cell clones. Mammalian expression vector constructions have been used to express genes in human cells. Lists of mammalian expression vectors can be found in a variety of texts and manuals about recombinant DNA techniques, gene transfer, and/or gene therapy. Expression vectors determine the expression level and the quality of recombinant mAbs [1, 2].

Previous investigations of the co-transfection using two monocistronic heavy chain (HC) and light chain (LC) expression plasmids studied the influence of constructs on the folding and the assembly of IgG in mammalian cells [3, 4]. However, further it was demonstrated that a vector containing a bicistronic construct was more efficient for the production of IgG in mammalian cells [5].

The use of internal ribosome entry sites (IRESs) provided a new tool for co-expressing multiple polypeptide chains of oligomeric or oligosubunit proteins in polycistronic expression systems [6].

Studies of Mizuguchi et al. compared the expression of IRES-dependent second gene with the cap-dependent first gene one in a bicistronic vector in several cell lines. These findings showed expression of the IRES-dependent second gene from 20 to 50% of that of the first gene [7]. Some experiments also showed that the orientation of promoters and the presence of introns affect the expression of immunoglobulins. In addition, the optimal results were demonstrated when cells were transfected with the expression plasmid that contained introns and had a head-to-tail direction of transcription [8, 9].

Various mammalian cell lines producing the recombinant antibodies are important for biopharmaceutical production. Overexpression and production of recombinant mAbs were studied using different vector constructions and can be achieved by transient or stable expression in different Chinese hamster ovary (CHO) cell lines as CHO-S, ExpiCHO, and CHO DG44 [10].

CHO cells are the most available among the numerous cell lines for the therapeutics mAb production [11, 12]. Rapidity and economy of the method are advantages of CHO cell transient transfection, which could therefore streamline the process of therapeutic drug development.

Transient expression systems are also very useful for studying the regulation of gene expression or for receiving experimental results in a short time frame. However, numerous recombinant proteins and antibodies used for preclinical or clinical trials were developed in stable transfected CHO DG44 and cultivated in a bioreactor [13].

The use of CHO cell expression systems, such as dihydrofolate reductase (DHFR) or glutamine synthetase (GS) [14, 15], encourages the advantages for further improvement of mAb production. Top growth rate in serum-free medium combined with high-expression recombinant protein cell lines allows to use CHO cells in large-scale production [13, 16, 17]. The influence of cell line lineage and media composition on productivity are well investigated; however, little is known about the metabolic features. Dean and Reddy [18] compared results from high-performer cell lines to a low-performer with high lactate-producing cell line that exhibits poor growth and productivity, and determined intrinsic metabolic profiles linked to their growth and production phases. Altamirano and DeBerardinis [19, 20] investigated that CHO cell culture is capable to consume nutrients like glucose and glutamine beyond their needs and secrete the enlarged waste products as lactate and ammonia.

Moreover, it was determined that these nutrients can be rather used for the synthesis of precursors of recombinant proteins than for cell growth [21–23]. It is known that limiting their secretion improves cell growth and performance in CHO culture [24].

We have constructed a few variations of expression vectors for the production of recombinant mAbs. Previous bipromoter and new bicistronic expression vectors with different arrangements were compared for further stable transfection. Transient gene expression successfully allows choosing the expression vector construction in this early stage of the process. At first, IgG expression levels were evaluated in transient transfection of CHO-DG44 cells. Development of a high-producing stable cell line for therapeutics monoclonal mAb production requires screening hundreds of clones. However, the highly productive cell line needs to possess low metabolic activity in order to have high longevity and productivity. Furthermore, the antibody expression level and the level of metabolism were previously analyzed in stable monoclonal cell clones. Thus, stable cell lines were developed by the combination of two approaches: analyses of the level of metabolites and the productivity of stable clones. Traditional antibody therapeutics production is time-consuming and some steps have to be optimized. New strategy of two approaches combined can be useful for ramping up therapeutic antibody development.

## Methods

### Plasmid construction

Bicistronic plasmids were generated using widespread commercial pOptiVEC™-TOPO® vector (Thermo Fisher Sci., USA). The bicistronic and bipromotor vectors were constructed for model anti-human-INF-beta mAb (production in CHO DG44 (*dhfr* −/−) cells.

All plasmids were constructed using standard cloning methods. Cloning procedure used the competent cells of *E. coli* strains XL1_Blue (a,b 3 recA1 endA1 gyrA96 thi_1 hsdR17 supE44 relA1 lac [F' proABlacIqZΔM15 Tn10 (Tetr)]) (Stratagene, USA) following standard protocol. The pOptiVEC™-TOPO® vector was modified by MluI restriction endonuclease recognition site upstream CMV promoter and polylinker into TA site for creating a universal expression vector. Modified expression vector can be usable both for transient and stable transfection for different CHO lines. The bicistronic and bipromotor vectors were constructed for further mAb production in CHO DG44 (*dhfr* −/−) cells.

DNA sequencing was performed to confirm the correct sequence following all cloning.

### Oligonucleotide primers design

Those specific oligonucleotide primers were designed.

- EuLA1Flid (5′-ATTAGCTAGCGACCAAAATTCAAAGACAAAATGG-3′)
- 272RvL (5′-CAGCCACAGTCCGTTTTATTTCCAACT-3′)
- EuHPA272Flid (5′-CCACCGCTAGCTCTTTGAGGAAAACAGGGTGTTG-3′)
- H-STApaI (5′-CGATGGGCCCTTGGTGGAGGCTGAG-3′)
- 272FcL (5′-GGAAATAAAACGGACTGTGGCTGCACC-3′)
- EMCVrev (5′-GCCTTATTCCAAGCGGCTTCGG-3′)
- IRES-R (5′-CCCGCTAGCTGTGGCCATATTATCATCGTGTTTTTCA-3′)
- IRES-F (5′-CCCGAATTCTAAGGGTTGGATCCCTACCGGTGCTG-3′)
- CMVfor (5′-CGCAAATGGGCGGTAGGCGTG-3′)

The primers were synthesized by and purchased from Evrogen, Russia (Moscow, Russia). After PCR assembly, resulting DNA fragments were amplified, purified, and cloned into a suitable vector. Resulting plasmid sequences were confirmed by DNA Sanger sequencing.

### Cell culture and cell lines transfection

CHO DG44 cell lines deficient in the dihydrofolate reductase gene (dhfr^-^) were obtained for Invitrogen (Thermo Fisher Sci., USA). The cells were kept in CO_2_ incubator with 98% humidified atmosphere and 8% CO_2_, at 37 °C.

### Media and cell cultivation

The initial cell density for plating was 3–4 × 10^5^ cells/mL, and viability was >99%. The cell cultures were subcultured after 2 days to maintain a high cell density and viability. Cell cultures were maintained in six-well plates and T-125 shaker flasks according to experiments. We used IMDM supplemented with 8 mM L-glutamine, 10% dialyzed fetal bovine serum (DFBS, Gibco, Thermo Fisher. Sci., USA) for transfection procedures. Cell lines were cultured in CD OptiCHO (Invitrogen, Thermo Fisher Sci., USA) supplemented with 8mM L-glutamine, 0.1% Pluronic F68, and antibiotic–antimycotic solution (all Gibco, Thermo Fisher Sci., USA) in static cell culture experiments.

Samples of cell cultures were centrifuged, and the supernatant was collected for further LDH assay, ammonia assay, and enzyme-linked immunosorbent assay (ELISA).

### Monitoring of cell growth

Viable cell density (VCD) and viability were measured in duplicates with using the trypan blue exclusion method with Goryaev chamber. Cell viability was calculated as a percentage of surviving cells compared to the total cell count.

### Transient transfection

Exponentially growing culture of wild-type CHO DG44 cells (Thermo Fisher Sci., USA) were seeded in a density of 3–4 × 10^5^ cells/ml in Iscove’s modified Dulbecco’s medium (IMDM) supplemented with 10% DFBS, 8 mM L-Glutamine and 1x Hypoxantine-Thymidine (HT) supplement (Thermo Fisher Sci., USA) in a six-well plate (Greiner BioOne, Germany). Cells were transfected at 70–80% monolayer confluency. Basal IMDM also was used for each of the experiments as the negative control. 1.0×10^6^ cells were transfected with 2 μg of supercoiled plasmid DNA in medium volume of 3 mL using a Lipofectamine -3000 Transfection Kit (Invitrogen, Thermo Fisher Sci., USA) following the standard Protocol. The cells were incubated for 4 h with a transfection mixture. The medium was then replaced with fresh IMDM medium supplemented with 10% DFBS, 8 mM L-glutamine, and without HT, and the cells were incubated for another 24–48 h.

The cells cultivated only in IMDM medium with 10% DFBS, 8 mM L-glutamine, and HT solution without Lipofectamine Reagent and pDNA were used for each of experiments as the control. The probes of supernatant were collected on 24 and 48 h after transfection.

Cells were transfected with 4 plasmid variants: pBiPr-HL–hh, pBiPr-HL–ht, pOpti-L-IRES(Short)-H, pOpti-L-IRES(Long)-H. Supernatants were collected at different post-transfection time points: 24–48 h. Antibody production was measured by ELISA.

### Stable transfection

Stable cell line was performed using linear pOpti-L-IRES(Long)-H according to the transient transfection results. The transfection of wild-type CHO DG44 cells (Thermo Fisher Sci., USA) and the resulting stable cell line producing IgG1 antibodies were represented using the Lipofectamine 3000 (Thermo Fisher Sci., USA) in a 6-well plate following the standard procedure. The day prior to transfection, cells were replated to a density of 0.4–0.5× 10^6^ cells/mL in basal IMDM (PanEko, Russia) supplemented with L-glutamine (Thermo Fisher Sci., USA), 1x HT supplement, and antibiotic–antimycotic solution (Thermo Fisher Sci., USA) in a CO_2_ incubator at 37°C with 96% humidity in an atmosphere of 8% CO_2_. After 24h of transfection, the HT-supplemented medium was withdrawn, and cells were cultured in a medium without HT at least for 5d. The stable cell lines were derived from transfected cells by CD OptiCHO/methotrexate (MTX) subsequent selection according to the standard manufacturer’s protocol. Clones were isolated by ClonePix Fl technology (Molecular Devices, USA) for selecting high-value clones and then plating into 96-well plates for 21 days. The productivity of clones stable cell lines was determined on days 7–9 of static T-125 shaker-flask cell culture and measured using ELISA following the standard protocol.

### Analytical methods for cell culture samples

#### Sample analyses

Viable cell density (VCD) and viability were measured in duplicates with using the trypan blue exclusion method with Goryaev chamber. Cell viability was calculated as a percentage of surviving cells compared to the total cell count.

Viable cell count (VCC) was determined using the following formula:

$$\textrm{VCC}=\frac{N_v}{N}\times 100$$

where VCC is viable cell concentration, *N*_*v*_ is the amount of unstained cells, and *N* is the total number of cells in 15 squares of Goryaev chamber.

Cell concentration was performed using the trypan blue dye exclusion method with a hemocytometer counting chamber and inverted microscope.

Total cell density (TCD) was calculated using the following formula:

$$\textrm{TCD}=N\times 1.1\times {10}^5$$

where TCD is the total cell density (cell/mL) and *N* is the total number of cells counted in 15 squares of the Goryaev chamber.

#### ELISA

The concentration of expressed mAbs in the culture supernatant was measured using enzyme-linked immunosorbent assay (ELISA). The level of IgG expression in the cell culture was determined using sandwich ELISA following the standard protocol. Mouse mAbs against light kappa-type chains of human Igs (Bialexa, Russia) were added to wells of a 96-well plate at a concentration of 0.5 μg/well. In the analysis, we used goat anti-human IgG (whole molecule) conjugated to horseradish peroxidase (Sigma, USA). IgG from human serum (Sigma, USA) was used as the standard. Duplicate well assay was used for both standard and sample testing. Samples were diluted in PBS buffer and loaded into a 96-well microtiter plate. Depending on the experiment, IMDM supplemented with 10% DFBS or CD OptiCHO were used in the experiments as the negative control and PBS buffer was used as the blank control. Unknown values were extrapolated against a linear regression of the human IgG standard absorbance versus concentration. The absorbance of samples at 450 nm was measured in each well using the ELISA microplate reader BioRad-680 (Bio-Rad Laboratories, Inc. USA).

#### Determination of LDH enzyme activity

Lactate as the metabolite of anaerobic glycolysis can be measured indirectly by using the activity of lactate dehydrogenase (LDH). The release of LDH was measured using a commercially available LDH Assay kit (Lactate Dehydrogenase Activity Assay Kit, Sigma). NADH was used as control. The intensity of the produced color was then measured colorimetrically at a wavelength of 450 nm using a spectrophotometer. The LDH activity of samples was determined using the following equation:

$$\textrm{LDH}\ \textrm{activity}=B\times \textrm{Sample}\ \textrm{dilution}\ \textrm{factor}/\left(\textrm{reaction}\ \textrm{time}\right)\times V$$

where B is the amount (nmole) of NADH generated (*T*_initial_ −*T*_final_), *V* is the volume.

#### Determination of ammonia level

The total level of ammonia or ammonium ion was measured by using a commercially available Ammonia Assay kit (Abcam, UK). The chemical equation that drives the relationship between ammonia and ammonium is:

$$\textrm{NH}3+\textrm{H}2\textrm{O}\leftrightarrow \textrm{NH}4++\textrm{OH}-$$

Ammonium chloride (NH4Cl) was used as standard. Samples were measured immediately on a colorimetric microplate reader at OD 570 nm. Concentration of ammonia and ammonium in nmol/μL (mM) in the test samples was calculated as:

$$\textrm{Ammonia}\ \textrm{and}\ \textrm{ammonium}\ \textrm{concentration}=\textrm{BV}\ast \textrm{D}$$

where *B* is the amount of ammonia and ammonium in the sample well calculated from standard curve in nmol. *V* is the sample volume added in the sample wells (μL) and *D* is the sample dilution factor.

## Results

### Construction of the expression vector for mAb production in CHO DG44 cells

Here, we have optimized the commercial pOptiVEC™-TOPO® vector by MluI restriction endonuclease recognition site upstream human cytomegalovirus (CMV) immediate-early promoter/enhancer for high-level gene expression in a wide range of mammalian cells and polylinker into TA site and further used them as expression vector. Based on previous investigations, light chain cassette was placed first downstream CMV promoter.

**Fig. 1:**
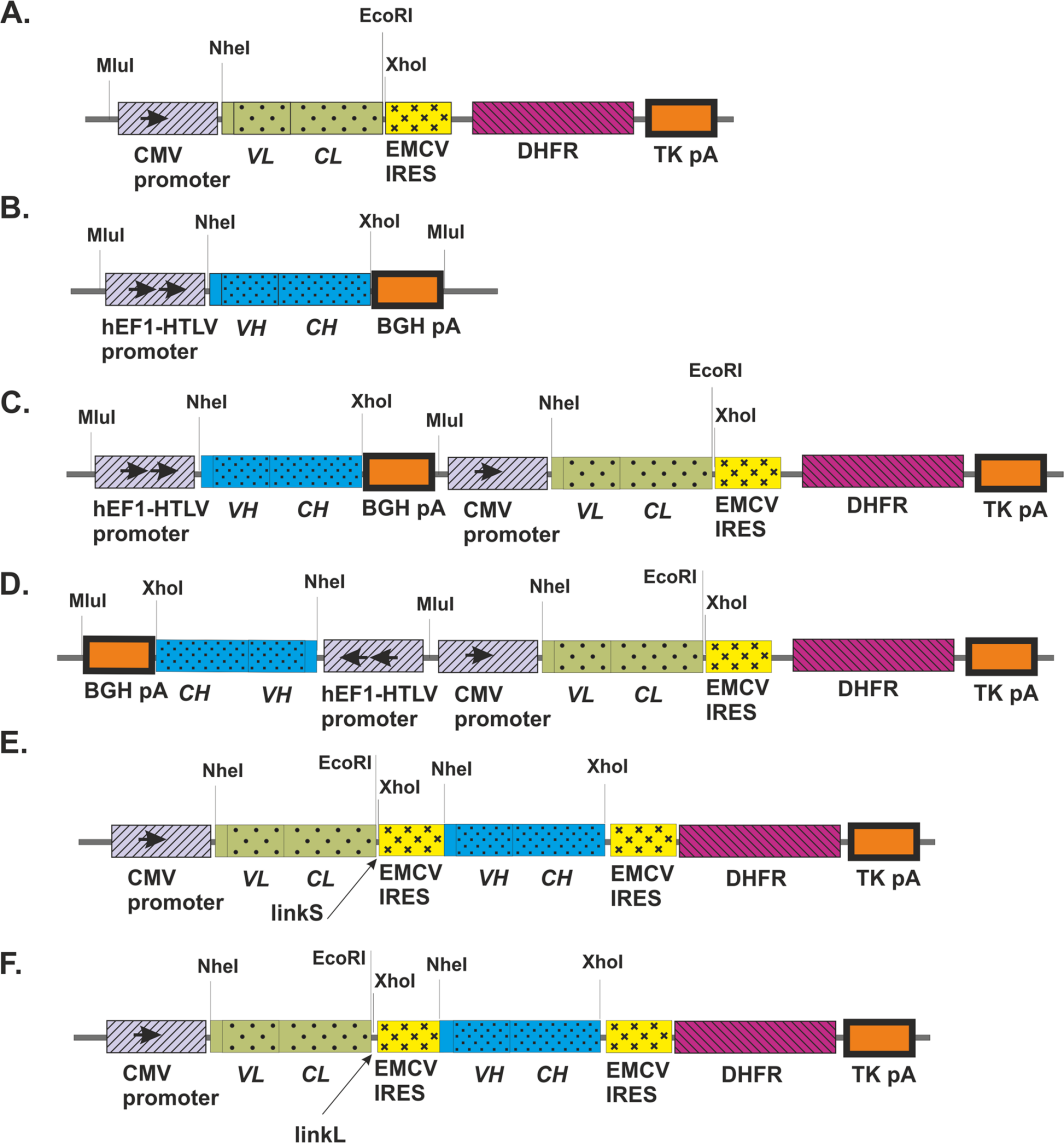
Schematic representation of the plasmid vectors pOpti-L-MluI (**A**), pSK-EF1-H-BGH (**B**), pBiPr-HL–ht (**C**), pBiPr-HL–hh (**D**), pOpti-L-IRES(Short)-H (**E**), and pOpti-L-IRES(Long)-H (**F**). VH and VL, variable domain coding sequences of HC and LC of the mAb with own Kozak sequences and leader peptides, respectively; CH and CL, constant domain coding sequences of the HC and LC of the mAb, respectively; EMCV IRES, IRES from the encephalomyocarditis virus (EMCV) for cap-independent translation of DHFR and HC of the mAb; TKpA, the Herpes Simplex Virus thymidine kinase polyadenylation signal for proper termination and processing of the recombinant transcript; hEF1-HTLV promoter, hybrid composite promoter comprising the Elongation Factor-1α (EF-1α) core promoter1 and the R segment and part of the U5 sequence (R-U5′) of the Human T-Cell Leukemia Virus (HTLV) Type 1 Long Terminal Repeat2; LinkS, short variant EMCV IRES (distance 54 b.p. between TGA LC and EMCV IRES beginning); LinkL, long variant EMCV IRES (88 b.p.)

Bicistronic vector can be used as basic vectors for the development of mAb CHO cell lines with high productivity. Variable domain coding sequences of the heavy (VH) and light (VL) chains of the mAb with own Kozak sequences and leader peptides were amplified using the previously obtained plasmid vectors [25] as a template. Forward primers for PCR contained NdeI restriction endonuclease recognition site and reverse primers contained the sequences overlapping with the start of the constant domain of the human light kappa-chain and the CH1 constant domain of the human IgG1 heavy chain, respectively, at 5′-terminus (primers were used EuLA1Flid and 272RvL for VL, EuHPA272Flid and H-STApaI for VH). Constant domain coding sequence of LC was amplified using previously obtained plasmid vector pOpti-F10L-MluI [26] as a template and primers 272F CL and EMCVrev for CL. Further, coding sequence for the LC of the mAb was obtained by combining the variable domain with own leader peptide and constant domain of the LC of the human immunoglobulin. Splicing by overlap extension PCR (SOE-PCR) was performed using external primers (EuLA1Flid and EMCVrev for LC). The PCR-fragment contained NdeI and XhoI restriction endonuclease recognition sites on the 5′- and 3′-ends, respectively. The LC expression cassette was cloned in pOpti-F10L-MluI plasmid vector into the NheI/XhoI sites under the control of CMV promoter (Fig. 1A).

Further, coding sequence for the HC of the mAb was obtained by combining the variable domains with own Kozak sequence and leader peptide and constant domains of the HC IgG1. PCR-fragment containing coding variable domain sequence of the HC with own Kozak sequence and leader peptide and NdeI and ApaI restriction endonuclease recognition sites on the 5′- and 3′-ends, respectively, was cloned into the NheI/ApaI sites in pSK-EF1-chimF10H-BGH [27] plasmid vector containing constant domains of the HC IgG1, hybrid promoter hEF1-HTLV, and bovine growth hormone polyadenylation signal (BGH polyA) (Fig.1B).

### Bipromoter vector design

For bipromotor vector construction, the MluI/MluI fragments contained coding sequence for mAb heavy chain under control of hybrid promoter hEF1-HTLV and BGH polyA was cloned into the MluI site in pOptiVEC-L-MluI plasmid vectors upstream mAb light chain coding sequence being under the control of CMV promoter (Fig. 1C and D). Two orientations of heavy chain gene cassette: head-to-tail (C) and head-to-head (D) bipromotor vectors were studied for mAb FI6-IgA production in CHO DG44 (*dhfr* −/−) cells [8].

### Bicistronic vector design

We developed a bicistronic design expression system which includes a VL-CL (1^st^ cistron) followed by VH-CН (2^nd^ cistron) under the control of a single CMV promoter.

For bicistronic vector design, the IRES from the encephalomyocarditis virus (EMCV) was used for cap-independent translation of DHFR and HC of the mAb. Two variants of EMCV IRES were amplified using pOpti-L-MluI as a template and primers 272FcL and IRES-R for long variant and IRES-F and IRES-R for short variant EMCV IRES.

Both PCR fragments contained EMCV IRES sequence flanked by EcoRI and NheI restriction endonuclease recognition sites on the 5′- and 3′-ends, respectively. The short variant EMCV IRES (link 1) had 54 b.p. between TGA LC and EMCV IRES beginning as it is recommended for pOptiVEC. The long variant EMCV IRES (link 2) had 88 b.p. between TGA LC and EMCV IRES beginning as it was in pOpti-F10L-MluI. The EMCV IRES EcoRI/NheI fragments and NheI/XhoI fragment from pSK-EF1-H-BGH contained expression cassette HC were cloned in pOpti-L-MluI plasmid vector into the EcoRI/XhoI sites downstream LC coding sequence under the control of CMV promoter (pOpti-L-IRES-H-MluI (short/long) as presented on Fig.1E and F.

### Analysis of IgG production levels for transiently transfected CHO-DG44 cells with four

**Fig. 2:**
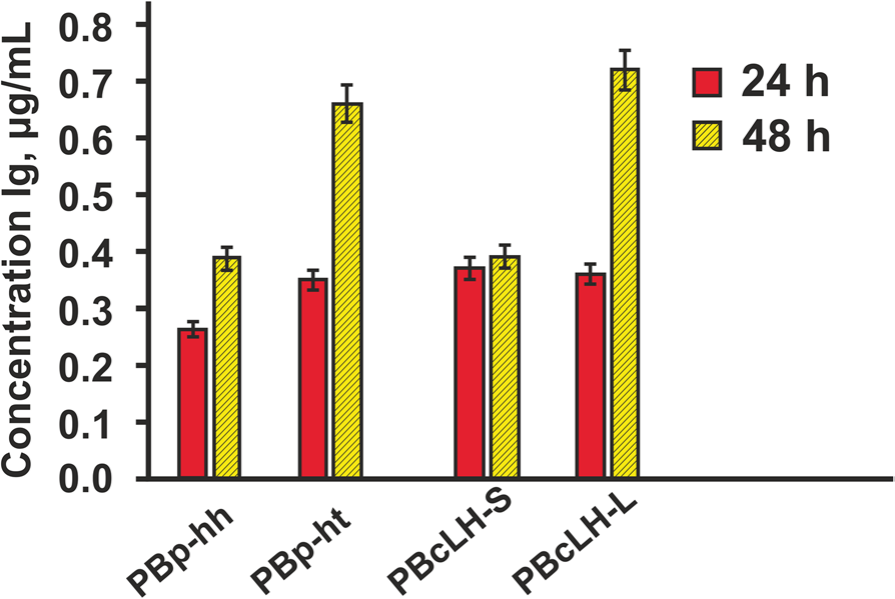
Сomparative analysis of the IgG expression level of different vector construction. Transient transfection in CHO-DG44 cells. Post-transfection time—24–48 h. Bipromoter vectors: PBp-hh (pBiPr-HL–hh), PBp-ht (pBiPr-HL–ht). Bicistronic vectors: PBcLH-S (pOpti-L-IRES(Short)-H), PBcLH-L (pOpti-L-IRES(Long)-H). Error bars depict standard deviations of the measurements

The IgG expression levels in the supernatant were measured by ELISA in 24–48 h after the transient transfection. Different combinations of expression constructs gave different expression levels. Maximum results were demonstrated when cells were transfected with the expression bicistronic plasmid that contained EMCV IRES-Long. The antibody expression level after transient transfection with the bicistronic plasmid pOptiVEC-L-IRES(Long)-H was 0.72 μg/ml, whereas the antibody expression level for the pOptiVEC-L-IRES(Short)-H variant of the plasmid reached 0.39 μg/ml that generally gave about 2-fold higher IgG expression than L-EMCV-IRES-Short-H construct at 48 post-transfection time (Fig. 2).

### Development of stable cell lines

#### The primary selection of high-value stable cell clones

Based on the transient expression data, the bicistronic expression plasmid with long link variant EMCV IRES was stably transfected into CHO-DG44 cells for the possibility to generate stably transfected high-expressing cell pools and further stable cell line development. After transfection, pool of transfectants was selected by ClonePix Fl technology/limiting dilution method for cell growth, high productivity, and monoclonality. The preliminary screening is usually an ELISA assay to eliminate non- or low producers. The productivity of high-producing stable clones was determined on day 10 of static six-well plate cell culture and measured using ELISA following the standard protocol. As demonstrated in Table 1, the highest antibody-expressing clones were 5B11 and 7D2 produced 40.0 and 42.0 mg/ml, respectively.

**Table 1:** Evaluation of IgG production in stably transfected CHO DG44 clones

	Clones				
	4D10	7G8	5B11	7D2	8F4
**Concentration of Ig, μg/ml**	**37.0**	**33.0**	**40.0**	**42.0**	**36.5**

### Metabolite characteristics for stable clone selection

**Fig. 3:**
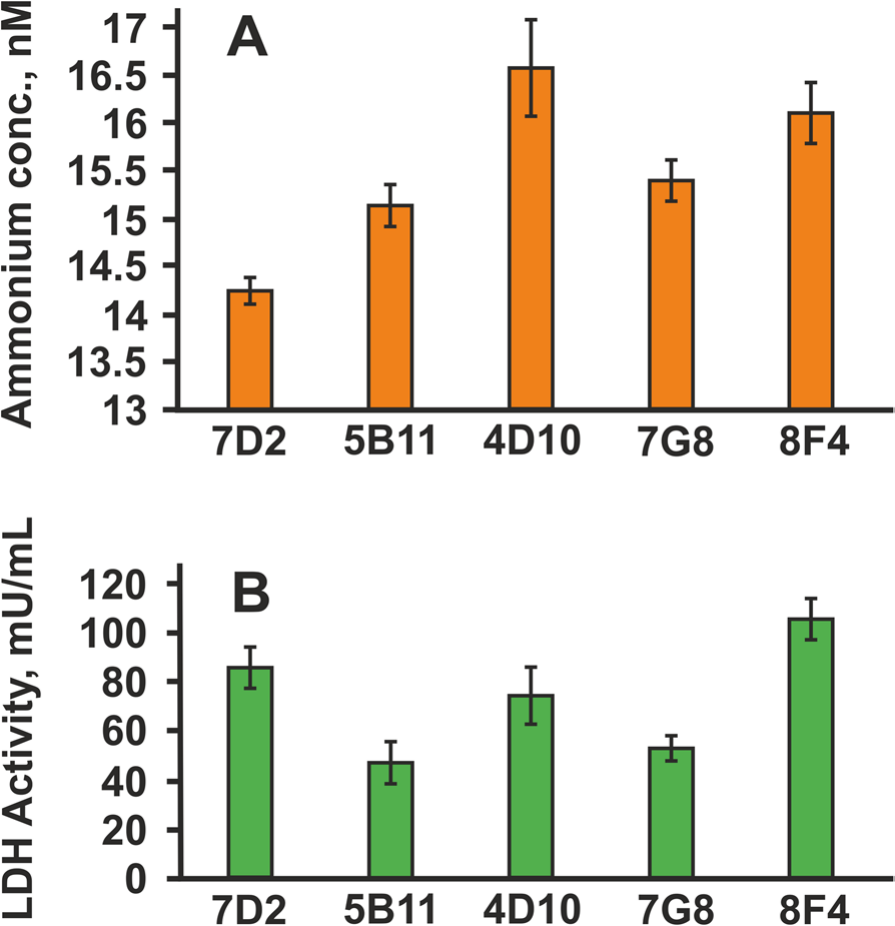
Influence of lactate and ammonia concentration on each stable cell clone selection. **A** Level of ammonia concentration for each stable cell clone on 10 days of static culture. **B** LDH activity profiles of stable cell clones on 10 days of static culture

In addition to the cell analysis and mAb production, we also analyzed the cellular metabolism by measuring key metabolites in supernatants at the end of the cultures for 10 days (Fig. 3).

The level of ammonium and lactate was determined on day 10 of same static six-well plate cell culture and measured following the standard protocol. The clones were selected based on the combination of the lowest levels of ammonium and lactate. As shown in Fig. 3, clone 5B11 demonstrated a small level of ammonium—15.1 nmol (A), and the lowest level of LDH—47.1 milliunits/ml (B). It can be noted that clone 7D2 had high lactate values 85.5 milliunits/ml but the lowest ammonium level—14.2 nmol. Since these data of clone 7D2 combined with the highest rate of productivity, this clone also was selected for further subcloning procedure.

**Fig. 4:**
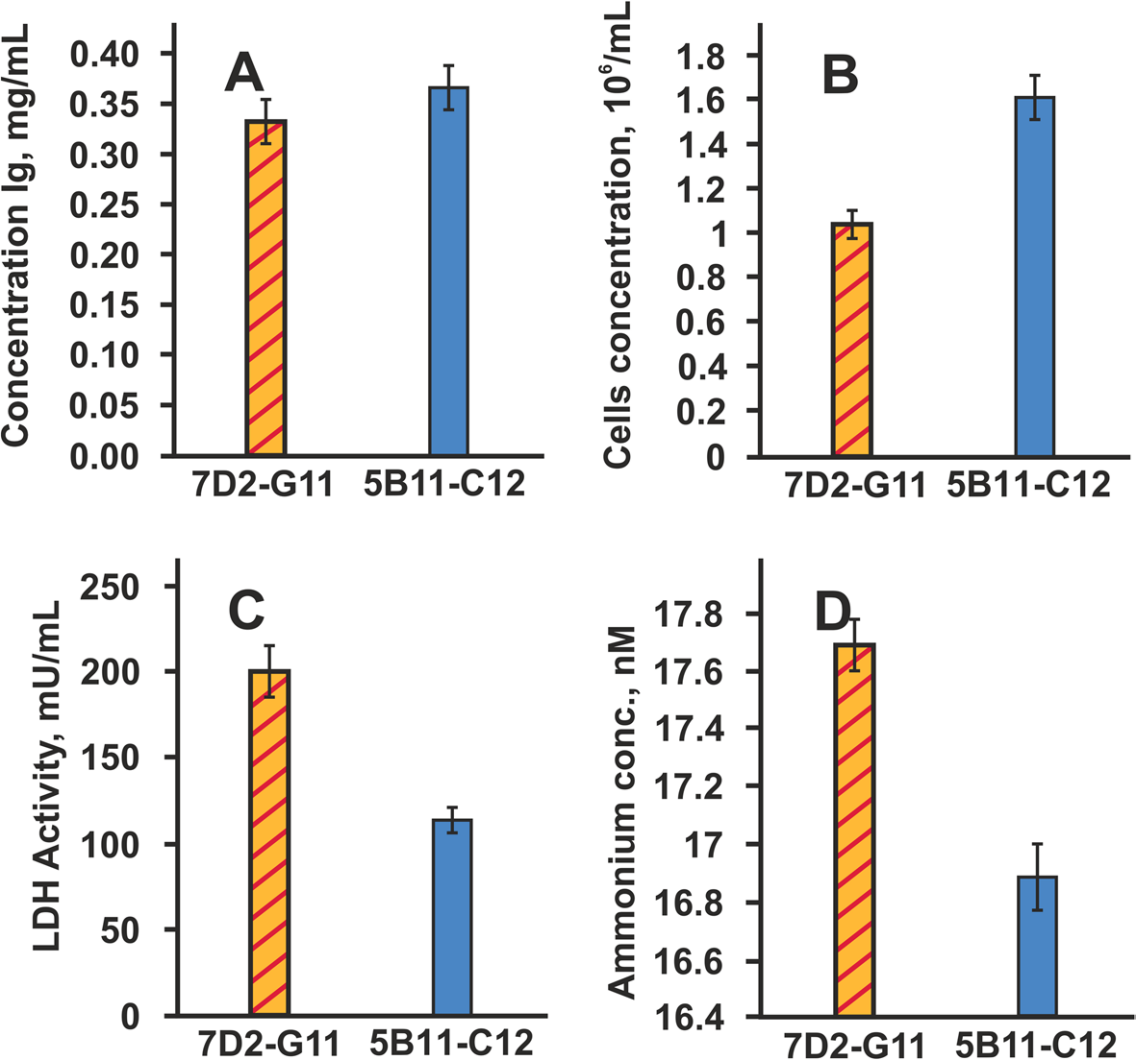
Selection of subclones for developing stable cell lines by IgG production, cell concentration, and metabolic selection. IgG productivity was determined by ELISA. Level of lactate production and ammonia concentration for selected subclones 5B11/C12 and 7D2/G11was determined by commercial kits

Additional steps are performed to measure cell growth, productivity, and metabolic characteristics in order to choose and compare the top two subclones candidates on second stage of selection. Clones 7D2 and 5B11 were subcloned by limiting dilution method. Changes in cell density, IgG concentration, LDH activity, and ammonium level were analyzed for each isolated subclone on second stage selection. As shown in Fig. 4, best cell concentration and productivity—1.56×10^6^ c/ml and 0.36 mg/ml, respectively, was detected for clone 5B11-C12 compared to clone7D2-G11 (Fig. 4A, B). Low metabolic data (lactate level 114.6 milliunits/ml and ammonium 16.8 nmol) was also demonstrated for subclone 5B11-C12 (Fig. 4C, D).

**Fig. 5:**
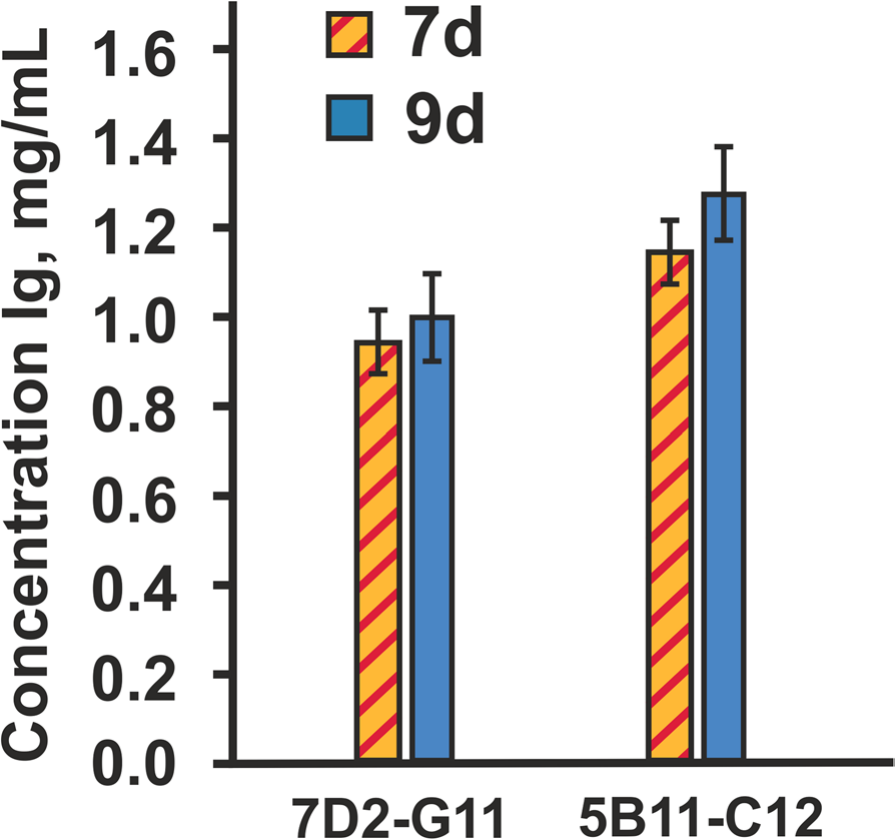
Comparison of the static shake cultures of selected stable cell lines on 7–9 days. IgG productivity was determined by ELISA

These two subclones were chosen for scaling as cell lines culture and then transferred into shake flasks for a second, larger-scale evaluation that is more representative for the final mAb productivity. Two cell lines PB-5B11-C12 and PB-7D2-G11 were adapted to cultivation in T-125 shaker flasks in serum-free medium and grown in static culture up to 10 days. We measured the IgG production throughout the shake flask study. As shown in Fig. 5, the production of IgG1 for cell line PB-5B11-C12 exhibited an increase of 22% compared to the cell line PB-7D2-G11 on day 9.

The IgG production in the stable cell line PB-5B11-C12 established from selected subclone 5B11-C12 was higher compared to the other subclones and a stable cell line was developed approximately 2 times faster than that of the standard procedure.

## Discussion

In the process of creating bispecific antibodies, the problem of synchronous expression of two or more genes in eukaryotic cells using a single self-compatible expression vector can be arised. Also, this vector should be suitable both for transient transfection and stable cell line generation. This problem may be solved by a novel bicistronic vector design. mAb against human INF-beta was used as a model of protein expression. Transient gene expression is a methodology employed in bioprocessing for the fast development of recombinant IgG production. To compare the produced plasmid constructs, we used four types of expression plasmids. Along with the effect of the promoter orientation, we also studied how the bicistronic construction affects the expression of the antibody versus to bipromoter variants constructions. Antibody expression level after transient transfection with the bicistronic plasmid was higher than with biplasmid vectors. This result was achived probably due to IRES-links in the plasmid construct. The stable transfection of cells is a longer process compared to transient transfection and designed for such research as recombinant immunoglobulin production on a large scale or longer-term pharmacology studies. Large amounts of recombinant Igs with proper folding and post-translational modifications are usually produced by stably transfected cell lines [28, 29]. Combination of two approaches allows to avoid the selection of stable cell clones with high level of ammonium and lactate that have low longevity as seen in traditional approaches of generating stable cell lines. Streamline of CHO-DG44-derived stable cell lines selection process was developed by a combination of two strategies: screening of productivity and level of metabolites. Stable transfected cells were monitored for growth, productivity, level of ammonium, and LDH.

At the next stage, in order to select stable cell clones with low level of metabolites, the same clone attributes should be considered and evaluated for features such as level of ammonium and lactate. Ammonium and lactate are a metabolic product, which is created through amino acid deamination and glucose metabolism [30]. After the second stage selection, the chosen subclones were transferred into shake flasks for scaling up of IgG production. Static shake cultures of selected stable cell lines were higher compared to the other subclones and a stable cell line was developed approximately 2 times faster than that of the standard procedure.

## Conclusions

We have developed a series of mammalian expression vectors for the production of recombinant monoclonal IgG antibodies with different expression cassette designs. In the current study, we used bipromoter and bicistronic expression plasmid constructs for the enhanced gene expression in CHO cells. Data analysis results of transient transfection in CHO cells showed that bicistronic expression plasmid design in CHO cells was better than previously reported results with bipromoter plasmid [8]. Our results indicated that compared to bipromoter expression, EMCV IRES-long link-mediated bicistronic expression constructs yield higher antibody expression levels, long-term stability, and advantage for generation of stable cell lines. In stably transfected CHO DG44 cells, bicistronic antibody expression with EMCV IRES-Long link in 88 b.p. gave the higher IgG yields than construct with short link in 54 b.p.

To test the long-term effect of the EMCV IRES-Long link element on mAb expression, stable CHO cell lines were generated with a bicistronic construct and analyzed for expression levels and stability. Usually limiting dilution cloning method and ClonePix procedure were performed for the clone screening and selection, which is relatively labor-intensive and time-consuming. To develop metabolic-efficient CHO cells from transfected heterogeneous pool, we applied a two-stage selection strategy. Implementation of a two-stage selection strategy allowed the elimination of low- and poor-producer clones in the early step of selection. Additional use of metabolic level intensity to estimate the IgG production allows screening numbers of transfectants more effectively than by only conventional expression tests such as ELISA. Application of the two strategies’ combination improves the chances of identifying high-producer clones and significantly reduces the effort required for the development of stable cell lines. One of the major advantages of this strategy is the ability to move quickly to large-scale mAb production by creating stable cell lines with low metabolic profiles using. To date, this is a significant improvement in the map development profile at the clonal cell selection stages and we expect further upstream process optimization.

## Data Availability

Not applicable.

## Abbreviations

CHO: Chinese hamster ovary cells
mAbs: Monoclonal antibodies
DHFR: Dihydrofolate reductase gene
HEK: Human embryonic kidney cells
IMDM: Iscove’s modified Dulbecco’s medium
DFBS: Dialysed fetal bovine serum
ELISA: Enzyme-linked immunosorbent assay
VCD: Viable cell density
VCC: Viable cell count
TCD: Total cell density
LDH: Lactate dehydrogenase
MTX: Methotrexate, antimetabolite of folic acid
NADH: Nicotinamide adenine dinucleotide reduced
VH/VL: Variable domain coding sequences of the heavy/light chains
CH/CL: Constant domain coding sequences of the heavy/light chains
SOE-PCR: Overlap extension PCR
CMV: Cytomegalovirus
BGH polyA: Bovine growth hormone polyadenylation signal
IRES: Internal ribosome entry site
TkpA: Thymidine kinase polyadenylation
hEF1: Hybrid elongation Factor-1α
HTLV: Human T-cell leukemia virus
